# Perceptual Decision Efficiency and Optimal Sleep Quality Are Associated with Female College Soccer Injury Avoidance

**DOI:** 10.3390/brainsci16060624

**Published:** 2026-06-11

**Authors:** Gary B. Wilkerson, Marisa A. Colston, Madison R. Ekas, MacKenzie L. Perkins, Rebecca L. Rinehart, Lynette M. Carlson, Jennifer A. Hogg, Shellie N. Acocello

**Affiliations:** 1 Department of Health & Human Performance, University of Tennessee at Chattanooga, Chattanooga, TN 37403, USA; marisa-colston@utc.edu (M.A.C.); lynette-carlson@utc.edu (L.M.C.); jennifer-hogg@utc.edu (J.A.H.); shellie-acocello@utc.edu (S.N.A.); 2 Graduate Athletic Training Education Program, University of Tennessee at Chattanooga, Chattanooga, TN 37403, USA; hzy499@mocs.utc.edu (M.R.E.); njd859@mocs.utc.edu (M.L.P.); drz188@mocs.utc.edu (R.L.R.)

**Keywords:** perceptual decision-making, perceptual learning, immersive virtual reality, injury prevention, sleep impairment

## Abstract

**Highlights:**

**What are the main findings?**

• Perceptual decision efficiency (PDE) estimated from eye responses to horizontally moving Virtual Reality (VR) visual stimuli appears to be highly modifiable through VR training that involves tasks of progressively increasing difficulty.
• Both optimal PDE and optimal sleep quality were associated with avoidance of concussion or a time-loss musculoskeletal injury affecting the lower extremity.

**What are the implications of the main findings?**

• Poor quality sleep may be a factor that adversely affects PDE, both factors may contribute to injury susceptibility, and either factor could be a consequence of a prior injury to some variable extent.
• Detection of a subtle impairment in perceptual decision making appears to require a task that imposes a sufficient level of difficulty, as well as a performance metric that optimally reflects components of the neural processes involved in generating rapid responses to alternative choices.

**Abstract:**

Background: Sport-related injuries are common, and often recurrent, among female college soccer players. This exploratory cohort study investigated whether perceptual decision efficiency and sleep quality could discriminate between injured and uninjured players. Methods: Twenty-seven NCAA Division I women’s soccer players (19.5 ± 1.3 years) completed a perceptual response training program, administered through an immersive virtual reality system, across a 13-week season. Players completed 11 training sessions progressing through four levels of task difficulty, with conjugate eye movements, neck rotation, and whole-body lunge-reach responses measured for each trial. Four metrics, elapsed time, rate correct per second, across-trials variability, and an efficiency index, were calculated for each of three defined time segments: perceptual decision, action initiation, and perceptual–motor response. The Pittsburgh Sleep Quality Index (PSQI) and Global Well-Being Index (GWBI) were administered prior to the first practice session, and all subsequent time-loss injuries were documented. Receiver operating characteristic analyses, Kaplan–Meier survival analysis, and classification tree modeling were used to evaluate injury discrimination. Results: Twelve time-loss injuries, including five concussions and seven lower extremity musculoskeletal injuries, were sustained by 10 of the 27 players. Optimal discrimination between injured and uninjured players was derived from the perceptual decision efficiency (PDE) metric for the most difficult perceptual response training task (AUC = 0.682–0.794), with a binary cut point of ≤6.02 yielding an odds ratio of 5.60 (95% CI: 1.02, 30.90; Mantel–Cox log rank *p* = 0.025). All five concussions occurred in players classified as high-risk by a suboptimal PDE value. Pre-participation PSQI demonstrated an AUC of 0.735. Notably, no player with both an optimal PDE value and a favorable sleep quality score (PSQI < 4) sustained a time-loss injury. Moderate-to-large training-related improvements in perceptual decision metrics were observed for the least challenging task from early- to late-season sessions. Conclusions: Optimal values for PDE and sleep quality together characterized female college soccer players who avoided injury. Both factors appear to be modifiable, suggesting that perceptual response training combined with interventions to enhance sleep quality may enhance injury resistance. Independent validation in larger, diverse athlete cohorts is warranted.

## 1. Introduction

The adage “An ounce of prevention is worth a pound of cure” has been attributed to Benjamin Franklin’s 1736 reference to the prevention of fires [1], but it equally applies to the maintenance of optimal human performance capabilities. Sport-related injuries often have cumulative adverse effects on performance that the natural healing process does not completely resolve, and persisting dysfunction cannot always be eliminated by therapeutic interventions. In fact, a history of injury is well-established as a strong predictor of future injury [2]. For example, the occurrence of a sport-related concussion (SRC) increases risk of a subsequent musculoskeletal injury [3,4,5,6,7], as well as the likelihood of another SRC [8,9,10,11].

Soccer participation presents substantial risk for injury, which occurs at a rate of 8.33 injuries per 1000 athlete-exposures among female players at the college level [12]. Approximately 65–70% of musculoskeletal injuries are lower extremity sprains and strains. The SRC incidence rate is 7.15 per 10,000 athlete-exposures for female players, which is 1.6 times greater than 4.43 per 10,000 athlete-exposures for male players [13]. Furthermore, a 12.5% rate of recurrent SRC among female college players is 3.4 times higher than the 3.6% rate for male college players [14]. Ball heading is a factor unique to soccer, which may produce subclinical pathological changes that elevate SRC risk [15,16,17]. Inadequate neck strength and slower responsiveness of female players could permit greater head acceleration, which would exacerbate this factor [15].

Although the exact mechanism responsible for recurrent injuries is not well-understood, impairment of brain processes that transform sensory inputs into effective muscle responses is believed to be a critical factor [18,19,20,21,22,23,24]. Persistent SRC effects can clearly increase musculoskeletal injury risk, and reverse causality also appears possible [25,26]. Early visual, auditory, or proprioceptive detection of an impending event that could result in injury is essential to initiate muscle responses for either impact avoidance or resistance to potentially injurious body segment displacements. For example, failure to visually detect an impending head impact may preclude rapid neck muscle stiffening, which would result in unrestrained head acceleration [27,28]. Any factor that may disrupt perceptual–motor processing in the brain, including the documented adverse effects of biopsychosocial distress on injury susceptibility [29], needs to be proactively identified and addressed.

Perceptual decision-making is increasingly recognized as an important factor associated with both soccer performance capabilities and injury avoidance [30], which is closely related to the control of sensorimotor interactions with the performance environment [31]. A recent frame-by-frame video analysis of non-contact anterior cruciate ligament tears among professional soccer players demonstrated evidence of poor decision-making in 91% (43/47) of the injury events [32]. Sleep quality is a factor believed to influence brain perceptual processing of sensory inputs [33,34,35,36], and a prospective association between disordered sleep and sport-related injury occurrence has been documented for both musculoskeletal injuries [37,38] and SRC [39,40]. Sleep disorders have been closely linked to emotion regulation [41] and mediation of the relationship between a history of multiple SRCs and depression symptoms [42,43]. Moreover, disordered sleep has been reported to have both an independent effect and a synergistic effect with stress that can activate a persistent neuroinflammatory response and reduce the efficiency of neural processing [44].

Despite concern that current clinical tests are inadequate for detection of subtle abnormalities in brain function after concussion [6,17,18,27], established behavioral correlates of key neural processes can be used to design screening and training tasks that may be more sensitive and feasible for administration in a clinical setting. Tests that require a choice between alternative responses have long been used to assess response time (RT) duration and response accuracy (RA), and a growing body of evidence strongly supports intra-individual variability (IIV) across successive test trials as an exceptionally good behavioral indicator of the changes in brain state required for the generation of fast, accurate, and consistent responses to sensory stimuli [45,46]. A variety of methods have been used to quantify RT as the time elapsed from presentation of a stimulus to a simple button press, arm reach for manual contact with a sensor, or whole-body displacement over a specific distance. Immersive virtual reality (VR) can be used to precisely quantify time intervals from the onset of a visual stimulus to response endpoints measured by an eye-tracking camera and sensors within the VR display headset, as well as sensors within hand controllers. This technology allows users to carry out tasks that engage cognitive processes, such as visual scanning, orienting of attention, and responding with actions toward objects in a controlled manner. A recent systematic review of the effect of VR training on the performance of soccer players concluded that it provides an effective approach to improve cognitive–motor skills, particularly those involved in decision-making under time pressure, and that they may be realized with as few as three training sessions and as little as five minutes per session [47].

Development of new strategies for the detection and remediation of suboptimal neural processes underlying perceptual decision-making clearly has the potential to provide profound benefits. The dominant hypothetico-deductive framework of scientific investigation maximizes the internal validity of its results, but rigorous control of potentially confounding factors can severely limit their applicability to real-world problems [48]. Data-driven machine learning offers an inductive approach to problem-solving that can maximize prediction accuracy for a pre-specified outcome through numerical optimality of algorithm components, but it has historically produced models that lacked explainability for real-world applications [49]. Another approach is exploratory data analysis involving iterative statistical procedures guided by domain knowledge to create a prediction model that will be readily interpretable and clinically meaningful. Thus, the purpose of this exploratory cohort study was to utilize insights derived from prior investigations to guide implementation of immersive VR training activities and data analysis procedures expected to yield an increased understanding of factors influencing the perceptual decision-making and injury susceptibility of female college soccer players.

## 2. Materials and Methods

### 2.1. Participants and Institutional Review Board Statement

A total of 27 college women’s soccer players (19.5 ± 1.3 years, 1.7 ± 0.05 m, 65.1 ± 7.3 kg) who comprised the entire roster of an NCAA Division I team participated in the study. As members of a collegiate team roster, each athlete completed a consent and authorization for access to protected health information for research purposes. All procedures were approved by the Institutional Review Board of the University of Tennessee at Chattanooga (#23-052 approved on 8 May 2023). The study complied with the Declaration of Helsinki (DoH)—Ethical Principles for Medical Research Involving Human Participants (1964) and its latest amendments adopted by the 75th General Assembly of the World Medical Association (WMA) in Finland on 19 October 2024. The only exclusionary criterion was an injury-related impairment that limited the ability to perform rapid arm reaching and single-step lunging movements.

### 2.2. Procedures

On the day prior to the first pre-season practice session, each player participated in an immersive VR familiarization session, and they completed well-validated surveys that included the Global Well-Being Index (GWBI) [29] and the Pittsburgh Sleep Quality Index (PSQI) [50,51] through the electronic REDCap system [52]. A subsequent injury was defined as a concussion or musculoskeletal injury that resulted in lost participation for at least one day beyond the date of injury, which was documented by athletic trainers across a 13-week period that included all practice sessions and 19 games. At least 2 PRT sessions, and as many as 11 sessions, were completed the previous season by 63% (17/27) of the players.

The PRT task presented horizontally moving visual stimuli on the immersive VR headset display (i.e., green circles with a filled green interior against a black background or green rings with an unfilled black interior), with instructions to execute either a right or left lunging and reaching body movement toward a designated response target (i.e., same direction as circle movement and opposite direction to that of ring movement), as described and illustrated in prior publications [53,54,55]. However, continued refinement of the PRT program produced modifications and added features to those previously reported, which are specified in a redefined Level 1 through Level 6 progression of task difficulty presented in Table 1. In addition to a requirement for visual scanning of both visual fields to locate and discriminate the target visual stimulus from a set that included 2 moving distractor stimuli, Level 6 imposed a working memory demand that substantially increased cognitive load.

**Figure 1:**
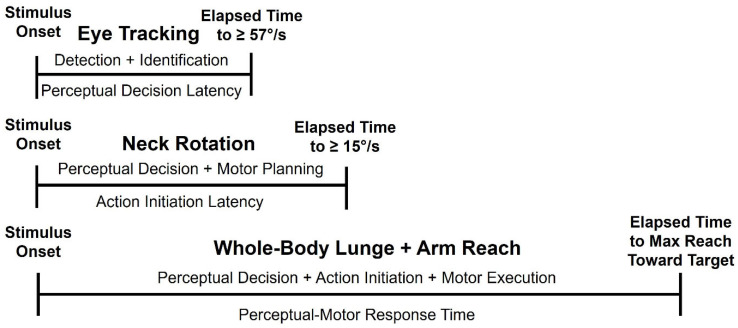
Operational definitions for time segments utilized to compute perceptual response training performance metrics for theoretical linkage to covert neural processes underlying perceptual decision-making.

Definitions of 3 different time segment endpoints also differ from those previously reported (Figure 1), with: (1) Perceptual Decision Latency derived from a velocity threshold of 57° per second (1 rad per second) for conjugate eye movement, (2) Action Initiation Latency derived from a velocity threshold of 15° per second for neck rotation, and (3) Perceptual–Motor Response Time defined by maximum hand controller displacement toward the response target located beyond the margin of the peripheral field of view and at a distance determined from a measurement of arm length to ensure that a lunge-step with arm reaching would be necessary to reach it. The virtual response targets disappeared when contacted, with a simultaneous auditory tone confirming a correct response execution.

A total of 40 trials were completed for each PRT session, and 4 performance metrics were calculated for each of the 3 time segments (i.e., Perceptual Decision, Action Initiation, and Perceptual–Motor Response): (1) elapsed time (ET; appearance of visual target(s) to time segment endpoint), (2) rate correct per second (RCS; number correct divided by total ET for 40 trials), (3) across-trials variability (ATV; intra-individual standard deviation for 40 trials), and (4) efficiency index (EI; RCS divided by ATV). The EI metric was deemed the most important, because its calculation includes components of the others to derive a single quantitative representation of the speed, accuracy, and consistency of responses.

Data for the single Level 2 familiarization session were not included in the analysis. Training sessions were planned for each week of the season, with Level 3 selected for a comparison of any improvements in performance metrics between initial and final sessions. After the initial Level 3 session, 3 consecutive weeks of training were completed at Level 4, as well as Level 5 and Level 6. The final session at Level 3 was planned for completion after a total of 10 previous sessions (i.e., the initial Level 3 session and 3 weekly sessions each for Levels 4–6). Although 7- day periods between sessions were planned, schedule adjustments to accommodate irregular team activities, such as travel to distant game locations, were anticipated.

### 2.3. Data Analysis

The Shapiro–Wilk test was used to assess the distribution normality of 12 PRT metrics acquired from initial and final training sessions at Level 3. Anticipating a mixture of normal and skewed distributions, the PRT data were analyzed by both dependent *t*-tests and Wilcoxon signed-rank tests. A *p* < 0.05 was interpreted as a finding likely to be replicated by future investigations of the same type, but null hypothesis significance testing was not among the purposes of this exploratory study. Effect size (ES) quantified the result of primary interest, with Cohen’s d derived from dependent *t*-tests (≥0.2 small, ≥0.5 medium, ≥0.8 large) [56] and Cohen’s r derived from Wilcoxon signed-rank tests (≥0.1 small, ≥0.3 medium, ≥0.5 large) [57]. Receiver operating characteristic analyses were used to identify the PRT metrics with greatest discriminatory value, with area under the curve (AUC) values ≥0.6 interpreted as providing potentially meaningful information [58]. Youden’s Index was used to define cut points for binary categorizations of high- versus low-risk status, with resultant odds ratio (OR) used to represent effect magnitude (≥1.32 small, ≥2.38 medium, ≥4.70 large) [59]. Logistic regression was used to evaluate potentially confounding effects among combinations of variables, but the cohort was too small for development of a multivariable prediction model. The binary categorization found to provide the best discrimination was subsequently used in a Kaplan–Meier time to event analysis. Bivariate correlation was used to assess possible associations among pairs of continuous variables and a classification tree was created to illustrate potentially meaningful relationships among the strongest binary predictors of injury occurrence. All analyses were performed using SPSS Version 29.0 (Armonk, NY, USA).

## 3. Results

The first Level 3 PRT session was completed 13 days after the start of pre-season practice sessions and the final session at Level 3 was administered 74 days later (i.e., an average of 7.4 days between sessions, with no inter-session interval shorter than 4 days or longer than 10 days). Among the four key metrics (i.e., ET, RCS, ATV, and EI) for each of the three defined time segments (i.e., Perceptual Decision, Action Initiation, and Perceptual–Motor Response) acquired at the beginning and the end the perceptual response training, 42% (10/24) of the Level 3 data distributions significantly deviated from normality, which are marked with asterisks in tables. Thus, pre- to post-training assessments of potentially beneficial training adaptations were performed with both dependent *t*-tests (Table 2) and non-parametric Wilcoxon signed-rank tests (Table 3). Moderate-to-large effect sizes were found for all four of the Perceptual Decision metrics derived from the Level 3 eye movements, with ATV and EI demonstrating the largest changes among all metrics.

A total of 12 time-loss injuries were sustained by10 of the players, which included five concussions. One player sustained both a concussion and a lower extremity injury (quadriceps) and another player sustained two lower extremity injuries (knee and lower leg). The other four injured players sustained a single lower extremity injury (three knee and one hamstring). The 12 injuries resulted in a total of 55 lost participation days. The best metrics for discrimination between the 10 injured players and the 17 uninjured players were Perceptual Decision EI values derived from the Level 6 sessions (AUC = 0.682–0.794), as well as the pre-participation PSQI score (AUC = 0.735). Median, inter-quartile range, and AUC values for each the Level 6 metrics are presented in Table 4.

Perceptual Decision Efficiency (PDE) represented by the EI metric incorporates each of the other metrics in its calculation, and it demonstrated a high degree of performance consistency over three sessions conducted within a 14-day period, with ICC (2,K) = 0.824. The PDE data derived from the first of the Level 6 sessions was chosen for analysis to exclude learning-related performance improvement after the first exposure to the most difficult VR task, which demonstrated a binary cut point of ≤6.02 and OR = 5.60 (95% CI: 1.02, 30.90). To assess the possibility of a differential training adaptation for PDE between injured and uninjured players, the Level 3 pre- and post-training values were analyzed by a group x trial repeated measures analysis of variance. A statistically significant and meaningful difference between trials (*p* = 0.001; η_p_^2^ = 0.343) was consistent with the results of the corresponding dependent *t*-test and Wilcoxon signed-rank test. There was a non-significant group x trial interaction effect (*p* = 0.643; η_p_^2^ = 0.009), and there was no significant difference between the groups (*p* = 0.474; η_p_^2^ = 0.021). Thus, both groups demonstrated a comparable amount of training-induced improvement.

**Figure 2:**
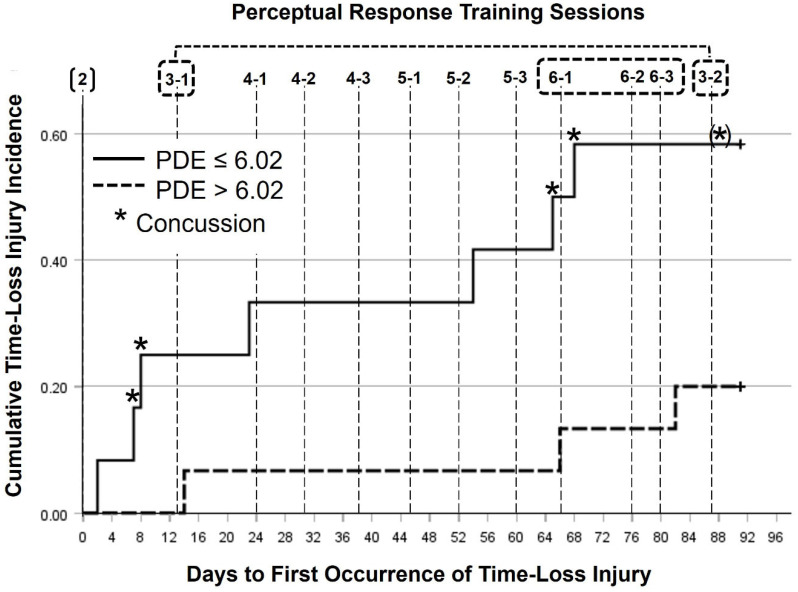
Kaplan–Meier time-to-event analysis for the first occurrence of a time-loss injury. The PDE ≤ 6.02 binary classification was derived from the first Level 6 training session (Mantel–Cox Log Rank *p* = 0.025). The asterisk enclosed by parentheses identifies a concussion occurrence that was preceded by a time-loss lower extremity injury. Vertical lines depict days corresponding to completion of perceptual response training sessions, with identification of their respective difficulty levels.

**Figure 3:**
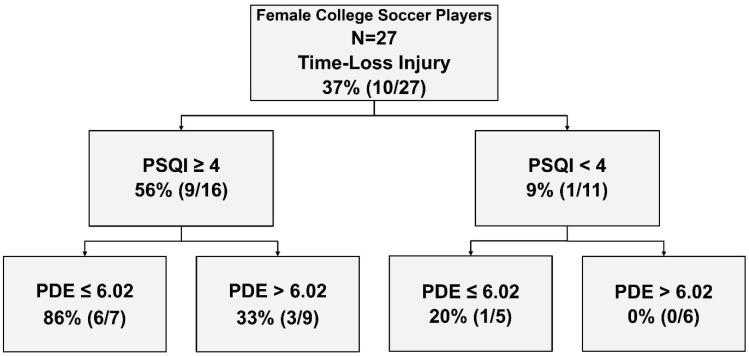
Classification tree for time-loss injury incidence among combinations of suboptimal (≥4) versus optimal (<4) scores for the Pittsburgh Sleep Quality Index (PSQI) and suboptimal (≤6.02) versus optimal (>6.02) values for the Perceptual Decision Efficiency (PDE) metric derived from the first training session completed at Level 6.

The results of a Kaplan–Meier time-to-event analysis for the PDE ≤ 6.02 binary classification derived from the first Level 6 training session demonstrated a Mantel–Cox Log Rank *p* = 0.025. All five concussions were sustained by players who were classified as high-risk on the basis of low PDE (Figure 2). The pre-participation PSQI demonstrated an AUC = 0.735, with high sensitivity (90%) and low specificity (59%). The combination of GWBI introspective distress ratings for sleep and mood disturbances demonstrated an AUC = 0.688, with low sensitivity (50%) and high specificity (88%). Further analysis of survey responses was limited to the more discriminatory PSQI, which demonstrated a potentially meaningful inverse linear relationship with Level 6 response accuracy for the 40 trials, with r = 0.520, *p* = 0.005. Due to the high likelihood of model overfitting, a multivariable analysis was not done. To illustrate a possible interaction between poor sleep quality and perceptual decision-making in highly demanding circumstances, a classification tree was constructed to depict time-loss injury incidence for player subgroups with a PSQI ≥ 4 cut point and the PDE ≤ 6.02 cut point derived from ROC analyses (Figure 3). There were no significant confounding effects for number of games played, number of minutes played in games, history of a time-loss core or lower extremity sprain or strain during the preceding 12-month period, lifetime history of concussion, or prior exposure to PRT during the preceding soccer season.

## 4. Discussion

### 4.1. Interpretation of the Study Findings

Our findings are consistent with those of previous reports that have linked eye movements to cognitive processes controlling perceptual decision-making [60,61] and sensorimotor integration [62]. Moreover, our results support the potential for perceptual learning through training that presents challenges of progressively increasing difficulty [63]. Importantly, effective differentiation between optimal versus suboptimal perceptual decision-making requires the imposition of a sufficiently difficult task to overcome a ceiling effect associated with relatively easy testing procedures [21]. Most, if not all, current clinical tests appear to be insufficiently sensitive to detect subtle neural processing impairments [17,64,65]. In addition to visual scanning for target stimulus identification, conflict resolution, distractor suppression, and choice of directional response, the PRT Level 6 task adds a working memory requirement that appeared to add substantial complexity to the decision-making process. Unsurprisingly, the Perceptual Decision performance metrics incorporating eye latency (i.e., ET, RCS, ATV, and EI) for the Level 6 task demonstrated the strongest discrimination between injured and uninjured players.

An important advantage of the PDE metric is its composite representation of both the inherent speed–accuracy tradeoff involved in rapid decision-making (i.e., RCS) and variability in the latency of eye movements across 40 trials (i.e., ATV). Intra-individual variability in behavioral performance has been identified as a possible indicator of a poor signal-to-noise ratio that reduces decision confidence [66], which may be exacerbated by lapses in attention. Attention fluctuates to a variable extent between stable/accurate and error-prone states, with focused attention better represented by low ATV than fast responses or high response accuracy [46]. An important link between observable behavioral variability and covert neural processing is the finding that infra-slow oscillations of the brain’s default mode network regulate integration versus decoupling within and between spatially separated populations of neurons [45,67,68]. Rapid changes among brain states have been shown to reflect interdependencies among the power law exponent of infra-slow neural frequencies, the complexity of higher frequency neural signals (i.e., sample entropy), and fluctuations in activation levels of different neuron populations (i.e., neural signal variability). Low intra-individual variability in behavioral performance (i.e., consistency) is inversely related to high variability of brain signals, which reflect rapidly shifting brain states that support cognitive flexibility [69,70,71,72].

Eye tracking metrics have been shown to reveal overt manifestations of covert neuronal activations involved in perceptual decision-making [61,73]. An eye saccade is a rapid and conjugate movement of both eyes to shift gaze position from one point in space to another, which can be initiated within 100 to 250 ms and can accelerate to a peak velocity as great as 700° per second [74,75]. Whether reflexive or voluntarily driven by a behavioral goal, saccade generation requires visual signal encoding and processing along a neural circuit through the occipital, parietal, and frontal cortices that ultimately activate the superior colliculus and oculomotor burst generator nuclei in the brainstem [73,75,76]. The ocular muscles must overcome inertia to accelerate the eye globes over 15° to 20° for the attainment of peak velocity [77]. The direction and amplitude are pre-programmed, such that the final gaze position cannot be changed after the saccade is initiated [78]. Consequently, a minimum of approximately 175 to 200 ms of pre-saccade cognitive processing is necessary to ensure a high level of response accuracy [73]. An antisaccade in the direction opposite to that of the target stimulus requires approximately 50 to 100 ms longer cognitive processing before initiation of the eye movement [75].

Saccade latency is defined by the time that elapses from the appearance of a target stimulus to the attainment of a specified velocity threshold, which distinguishes it from small-amplitude eye movements that are slower [75]. Under some circumstances, the features of a visual target can be identified without overt movement of the eyes, and small-amplitude mini-saccades can be generated during visual scanning [61]. Visual scanning performed by male soccer players to acquire relevant tactical information away from the ball location is generally completed within about 500 ms [79]. Visual search training has been reported to enhance covert target attention, optimize overt eye movements, decrease the number of saccades, and reduce overall scanning time [80]. Assuming a task-imposed choice between response targets located on opposite sides and outside the peripheral margin of the field of view, saccade latency (i.e., ET), accuracy (i.e., RCS), and trial-to-trial variability (i.e., ATV) can reasonably be considered to reflect aspects of the overall efficiency of the cognitive processing that generated the conjugate eye movements associated with a perceptual decision (i.e., PDE). From a functional standpoint, the speed, accuracy, and consistency of action initiation and completed execution of multi-segmental responses are critical for both sport performance success and injury avoidance. However, the PDE metric probably provides the best quantitative representation of the perceptual decision-making processes that ultimately determine the outcome of rapidly selected actions within a changing environment. Although the potential for transfer of perceptual training adaptations is controversial [81,82], evidence that perceptual learning changes gene expression related to both microstructural and functional connectivity within and between brain networks suggests that such adaptations are highly likely to provide some degree of transfer benefit in real-world circumstances [60].

Sleep disruption is a likely contributor to brain state instability that impairs perceptual decision-making [33], which is also closely linked to emotional states [44]. Associations between inadequate sleep and sympathetic nervous system activation are not well-understood, but excessive release of norepinephrine is believed to induce inflammatory gene expression and production of inflammatory cytokines [44,83]. Direct projections from the locus coeruleus of the sympathetic nervous system to the superior colliculus can modulate its generation of eye saccades, as well as its contributions to attention, gaze orientation, stimulus selection, distractor suppression, and integration of oculomotor and somatomotor programming for execution of goal-directed movements [84]. Optimal behavioral performance depends on an intermediate level of neuronal activation, but an excessive level of activation is associated with the generation of scattered noise from a broader area that interferes with the efficiency of information processing [85]. Specifically, the process of accumulating evidence from sensory inputs and working memory to support a perceptual decision becomes slower, less accurate, and/or more variable with sleep deprivation [33,34,36]. A mindfulness-based stress reduction program has shown to reduce symptoms of stress, anxiety, and depression [86], and to increase resting state functional connectivity between brain network nodes believed to support the maintenance of attention and information processing efficiency [87]. The PRT program we utilized could conceivably provide some degree of resilience to the adverse effects of poor sleep quality and psychosocial distress, and the Level 6 PDE metric might be useful for the detection of an optimal versus suboptimal state.

### 4.2. Limitations

The relatively small cohort size, as well as the inherent challenges to the control of extraneous factors surrounding a college women’s soccer team across its pre-season and in-season sport-related activities, must be considered in interpretation of our results. As an exploratory cohort study, there was no specification of hypotheses prior to its initiation, and adjustment of the alpha level for multiple comparisons was deemed inappropriate [88,89]. The discovery that the Level 6 PDE value was better than other PRT metrics for discrimination between injured and uninjured players cannot be considered a causative factor, as the first Level 6 training session was conducted after 70% (7/10) of the time-loss injuries (i.e., first occurrence for players who sustained more than one) had already occurred within 66 days of the start of pre-season practice sessions. Thus, a low PDE value could have been either the cause or the consequence of injury, or both. Whether a suboptimal performance reflected a pre-existing deficiency in perceptual decision-making or the effect of a recent injury, such an individual’s state of susceptibility to a future injury, should be recognized as a cause for concern. For 82% (14/17) of players who demonstrated an optimal PDE value and who did not sustain a concussion or time-loss lower extremity injury, the time point at which the first Level 6 value was acquired is probably not relevant to the value of having an objective estimation of perceptual decision-making ability that may be an indicator of a relatively lower level of susceptibility to future injury.

The large 30.90 upper limit for the 95% CI of the OR = 5.60 that discriminated uninjured from injured players was strongly affected by the small numbers of false-negative and false-positive cases (three and five, respectively). The CI is calculated on a logarithmic scale and then back-transformation to the original scale, which is a non-linear process that results in the upper limit being much further from the OR point estimate than the lower limit [90]. Despite this statistical effect, the relatively small cohort size imposes uncertainty about the true strength of the reported associations. Thus, the reported PDE and PSQI cut points should not be interpreted as well-validated clinical thresholds. Although test–retest reliability of metrics derived from the VR system have been reported to be good to excellent [91], an acknowledged conflict of interest and subsequent development of tasks that impose greater cognitive demand present a need for further independent validation of measurement accuracy and reproducibility.

This study’s greatest limitations cannot be easily reconciled. The difficulty of the Level 6 task was too great for successful completion without having first progressed from easier to harder levels. Thus, multiple training sessions would need to be administered before the start of a season to conduct a prospective analysis of Level 6 PDE association with subsequent injury occurrences. Because a typical college women’s soccer team consists of 20 to 30 players, more than one team would be needed to gain a substantial increase in statistical power for a larger study of this subpopulation of athletes.

The PSQI and GWBI surveys were completed on the day prior to the first practice session, but the impact of sleep quality and mood states on injury susceptibility were unlikely to have remained constant throughout the entire season. Repeated completion of the surveys was deemed to impose an excessive time burden on the athletes, whereas the investment of time in performance of the 11 PRT sessions offered the potential benefit of an increasing degree of injury resilience over time. Despite the possible influences of uncontrolled factors on internal validity, and the small number of female soccer players that comprised our cohort, the overall results clearly support the potential for improvement of sport performance capabilities and reduction in injury risk without any known adverse effects. Future large-scale studies are needed to confirm the PRT benefits that we documented.

### 4.3. Future Research

Approximately 50% of college athletes report daytime sleepiness from insufficient or poor-quality sleep [92], which presents a major opportunity for improvement of sport performance capabilities and reduction in injury risk. Independent validation of our findings is needed to confirm the associations of PDE and PSQI with injury avoidance in a much larger cohort of female college soccer players, or a large cohort of athletes representing a variety of sports and both sexes. A pre-participation assessment of PDE at the high level of demand imposed by Level 6 of the PRT training is needed to establish a prospective association with common sport-related injuries, including concussion. A better understanding of interactions among factors that increase susceptibility might be gained through longitudinal monitoring of data derived from a wearable device that provides an objective estimation of sleep quality and autonomic balance (i.e., heart rate variability). Such a multifactorial analysis might also include assessment of any benefit derived from concurrent participation in mindfulness training activities.

## 5. Conclusions

Immersive VR training activities designed to enhance perceptual decision-making and injury resistance produced substantial improvements in the PRT performance metrics of college women’s soccer players across a 13-week season. Among the players who attained a high level of eye movement efficiency during perceptual decision-making (PDE ≥ 6.02), only 18% (3/17) sustained a time-loss injury and none sustained a concussion. Among the remaining players with lower values, 70% (7/10) sustained a concussion and/or some other time-loss injury. Sleep quality (PSQI < 4) also appeared to support injury resistance, since no player with both high PDE and low PSQI sustained a time-loss injury (0/6). Among those with low PDE and high PSQI, 86% (6/7) sustained a time-loss injury either before or after first-time performance of a highly demanding VR task. Because both factors may be modifiable, the potential for some degree of increase in injury resistance among female college soccer players seems likely. Independent validation of our exploratory study findings is needed to justify the investment of time and resources necessary to achieve the related goals of performance enhancement and injury resistance.

## Figures and Tables

**Table 1:** Perceptual response training difficulty levels (restricted to Levels 3-6 for this study).

Level	Moving Visual Stimuli	Initial Location(s)	Task Elements
1	Single Circular Object: Filled Circle or Unfilled Ring 1.0 m/s R-to-L or L-to-R	Central No Movement Delay	Detect/Discriminate Resolve Conflict Respond Right or Left
2	Single Circular Object: Filled Circle or Unfilled Ring 1.0 m/s R-to-L or L-to-R	Central or Peripheral No Movement Delay	Detect/Discriminate Resolve Conflict Respond Right or Left
3	3 Circular Objects: Filled Circle or Unfilled Ring 1 Flashing Target Object 2 Non-Flashing Distractors 1.25 m/s R-to-L or L-to-R	Central or Peripheral Top = Distractor Middle = Target Bottom = Distractor No Movement Delay	Detect/Discriminate Resolve Conflict Respond Right or Left
4	3 Circular Objects: Filled Circle or Unfilled Ring 1 Flashing Target Object 2 Non-Flashing Distractors 1.5 m/s R-to-L or L-to-R	Central or Peripheral Target vs. Distractor: Top, Middle, or Bottom Movement Delay 0.25 s	Scan for Target Detect/Discriminate Resolve Conflict Respond Right or Left
5	3 Circular Objects: Filled Circle or Unfilled Ring 1 Flashing Target Object 2 Non-Flashing Distractors 1.5 m/s R-to-L or L-to-R	Central or Peripheral Target vs. Distractor: Top, Middle, or Bottom Movement Delay 0.1 s	Scan for Target Detect/Discriminate Resolve Conflict Respond Right or Left
6	3 Circular Objects: Filled Circle or Unfilled Ring 1 Flashing Target Object 2 Non-Flashing Distractors 1.5 m/s R-to-L or L-to-R Each Object Numbered	Central or Peripheral Target vs. Distractor: Top, Middle, or Bottom Movement Delay 0.7 s	Scan for Target Detect/Discriminate Resolve Conflict Remember Number Respond Right or Left to Matching Number

**Table 2:** Mean, standard deviation (SD), dependent *t*-test *p*-value, and effect size (ES: Cohen’s d) for pre-training to post-training Level 3 performance.

Time Period	Metric	Pre-Training Mean (SD)	Post-Training Mean (SD)	*p*	ES
Perceptual Decision (Eyes)	Elapsed Time (s)	0.323 * (0.071)	0.291 (0.038)	0.010	0.537
	Rate Correct per Second	3.16 (0.67)	3.51 * (0.55)	0.007	0.568
	Across-Trial Variability	0.125 (0.071)	0.063 (0.030)	<0.001	0.909
	Efficiency Index	40.42 * (34.66)	70.27 * (39.43)	<0.001	0.751
Action Initiation (Neck)	Elapsed Time (s)	0.497 (0.093)	0.520 * (0.124)	0.157	−0.280
	Rate Correct per Second	2.04 (0.41)	2.03 (0.46)	0.879	0.030
	Across-Trial Variability	0.137 * (0.056)	0.142 * (0.046)	0.610	−0.099
	Efficiency Index	18.00 (9.05)	16.09 * (7.34)	0.222	−0.241
Perceptual–Motor Response (Arm Reach)	Elapsed Time (s)	1.108 (0.155)	1.102 (0.143)	0.778	0.055
	Rate Correct per Second	0.90 * (0.14)	0.92 * (0.12)	0.284	0.210
	Across-Trial Variability	0.228 (0.110)	0.171 (0.057)	0.009	0.542
	Efficiency Index	5.04 (2.59)	5.87 (1.79)	0.095	0.334

* Shapiro–Wilk test of distribution normality *p* ≤ 0.05.

**Table 3:** Median, inter-quartile range (IQR), Wilcoxon signed-rank *p*-value, and effect size (ES: standardized test statistic divided by square root of N) for pre-training to post-training Level 3 performance.

Time Period	Metric	Pre-Training Median (IQR)	Post-Training Median (IQR)	*p*	ES
Perceptual Decision (Eyes)	Elapsed Time (s)	0.305 * (0.274–0.344)	0.297 (0.265–0.322)	0.006	0.529
	Rate Correct per Second	3.28 (2.76–3.65)	3.37 * (3.11–3.78)	0.005	0.541
	Across-Trial Variability	0.109 (0.070–0.187)	0.059 (0.036–0.077)	<0.001	0.741
	Efficiency Index	33.42 * (18.27–48.47)	58.95 * (43.16–83.14)	<0.001	0.707
Action Initiation (Neck)	Elapsed Time (s)	0.480 (0.418–0.567)	0.473 * (0.420–0.608)	0.264	0.215
	Rate Correct per Second	2.03 (1.75–2.39)	2.11 (1.64–2.39)	0.899	0.024
	Across-Trial Variability	0.126 * (0.090–0.176)	0.129 * (0.115–0.170)	0.400	−0.162
	Efficiency Index	17.16 (10.67–22.64)	16.36 * (10.79–19.66)	0.171	−0.263
Perceptual–Motor Response (Arm Reach)	Elapsed Time (s)	1.068 0.993–1.184)	1.089 (1.001–1.213)	0.990	0.002
	Rate Correct per Second	0.93 * (0.82–1.01)	0.92 * (0.82–1.00)	0.564	−0.111
	Across-Trial Variability	0.194 (0.145–0.293)	0.155 (0.130–0.192)	0.019	0.453
	Efficiency Index	4.86 (2.86–7.15)	5.61 (4.82–7.15)	0.136	0.287

* Shapiro–Wilk test of distribution normality *p* ≤ 0.05.

**Table 4:** Median, inter-quartile range (IQR), and receiver operating characteristic area under curve (AUC) values for 3 consecutive Level 6 sessions completed within a 2-week period (66 to 80 days after the first pre-season practice session).

Time Period	Metric	Session	Median (IQR)	AUC
Perceptual Decision (Eyes)	Elapsed Time (s)	1 *	0.471 (0.438–0.573)	0.644
		2	0.532 (0.435–0.568)	0.588
		3 *	0.498 (0.452–0.561)	0.753
	Rate Correct per Second	1	1.94 (1.57–2.17)	0.741
		2	1.83 (1.70–2.21)	0.682
		3 *	1.98 (1.74–2.10)	0.676
	Across-Trial Variability	1	0.307 (0.285–0.324)	0.671
		2	0.288 (0.265–0.304)	0.756
		3	0.268 (0.240–0.289)	0.741
	Efficiency Index	1 *	6.19 (5.51–8.41)	0.738
		2 *	6.77 (5.61–8.41)	0.682
		3 *	7.49 (5.95–8.81)	0.794
Action Initiation (Neck)	Elapsed Time (s)	1	0.595 (0.488–0.779)	0.694
		2	0.626 (0.490–0.737)	0.665
		3 *	0.606 (0.492–0.726)	0.647
	Rate Correct per Second	1	1.51 (1.17–1.96)	0.706
		2 *	1.48 (1.29–2.04)	0.676
		3	1.62 (1.38–1.90)	0.659
	Across-Trial Variability	1	0.309 (0.280–0.343)	0.585
		2	0.301 (0.280–0.347)	0.638
		3	0.255 (0.227–0.331)	0.709
	Efficiency Index	1	4.65 (3.58–6.99)	0.671
		2 *	4.71 (3.82–7.96)	0.676
		3	6.13 (4.47–8.31)	0.729
Perceptual–Motor Response (Arm Reach)	Elapsed Time (s)	1	2.244 (2.161–2.299)	0.612
		2	2.135 (2.058–2.228)	0.691
		3	2.121 (2.054–2.154)	0.729
	Rate Correct per Second	1 *	0.410 (0.388–0.439)	0.706
		2	0.449 (0.432–0.474)	0.718
		3	0.463 (0.446–0.484)	0.788
	Across-Trial Variability	1	0.271 (0.245–0.298)	0.426
		2	0.277 (0.224–0.306)	0.479
		3	0.253 (0.204–0.276)	0.547
	Efficiency Index	1 *	1.47 (1.38–1.68)	0.529
		2 *	1.57 (1.47–1.94)	0.612
		3 *	1.84 (1.71–2.13)	0.635

* Shapiro–Wilk test of distribution normality *p* ≤ 0.05.

## Data Availability

The data presented in this study are available from the corresponding author upon institutional approval. The dataset is not publicly available, due to an institutional restriction on the release of data. A specific request from an individual who possesses research credentials must be reviewed and approved.

## References

[1] Spee R.F., Kemps H.M., Vromen T. (2024). An ounce of prevention is worth a pound of cure. Neth. Heart J..

[2] Jacobsson J., Timpka T., Kowalski J., Nilsson S., Ekberg J., Dahlström Ö., Renström P.A. (2013). Injury patterns in Swedish elite athletics: Annual incidence, injury types and risk factors. Br. J. Sports Med..

[3] Fino P.C., Becker L.N., Fino N.F., Griesemer B., Goforth M., Brolinson P.G. (2017). Effects of recent concussion and injury history on instantaneous relative risk of lower extremity injury in Division I collegiate athletes. Clin. J. Sport Med..

[4] Harada G.K., Rugg C.M., Arshi A., Vail J., Hame S.L. (2019). Multiple concussions increase odds and rate of lower extremity injury in National Collegiate Athletic Association athletes after return to play. Am. J. Sports Med..

[5] McPherson A.L., Nagai T., Webster K.E., Hewett T.E. (2018). Musculoskeletal injury risk after sport-related concussion: A systematic review and meta-analysis. Am. J. Sports Med..

[6] Oldham J.R., Bowman T.G., Walton S.R., Beidler E., Campbell T.R., Smetana R.M., Munce T.A., Larson M.J., Cullum C.M., Bushaw M.A. (2024). Sport type and risk of subsequent injury in collegiate athletes following concussion: A LIMBIC MATARS Consortium investigation. Brain Inj..

[7] Woodrow J., Vohra A., Galal Y., Koolmees W., Lederman E., Erickson S., Shah A. (2025). From head to toe: Investigating postconcussion risks for lower extremity injuries in young athletes. Orthop. J. Sports Med..

[8] Abrahams S., Mc Fie S., Patricios J., Posthumus M., September A.V. (2014). Risk factors for sports concussion: An evidence-based systematic review. Br. J. Sports Med..

[9] Brett B., Kuhn A.W., Yengo-Kahn A.M., Solomon G.S., Zuckerman S.L. (2018). Risk factors associated with sustaining a sport-related concussion: An initial synthesis study of 12,320 student-athletes. Arch. Clin. Neuropsychol..

[10] Race M.K., Hahn-Ketter A.E., Spielman L.A., Selmanovic E., Sy K.L.T., Wellington R., Dams-O’cOnnor K. (2023). Traumatic brain injury history and baseline symptoms outweigh sex differences for risk of concussion in a sample of collegiate athletes. Brain Inj..

[11] Van Pelt K.L., Allred D., Cameron K.L., Campbell D.E., D’Lauro C.J., He X., Houston M.N., Johnson B.R., Kelly T.F., McGinty G. (2019). A cohort study to identify and evaluate concussion risk factors across multiple injury settings: Findings from the CARE Consortium. Inj. Epidemiol..

[12] Chandran A., Morris S.N., Boltz A.J., Robison H.J., Collins C.L. (2021). Epidemiology of injuries in National Collegiate Athletic Association women’s soccer: 2014–2015 through 2018–2019. J. Athl. Train..

[13] Chandran A., Boltz A.J., Morris S.N., Robison H.J., Nedimyer A.K., Collins C.L., Register-Mihalik J.K. (2022). Epidemiology of concussions in National Collegiate Athletic Association (NCAA) sports: 2014/15-2018/19. Am. J. Sports Med..

[14] Zuckerman S.L., Kerr Z.Y., Yengo-Kahn A., Wasserman E., Covassin T., Solomon G.S. (2015). Epidemiology of sports-related concussion in NCAA athletes from 2009-2010 to 2013-2014: Incidence, recurrence, and mechanisms. Am. J. Sports Med..

[15] Kakavas G., Malliaropoulos N., Skarpas G., Forelli F. (2025). The impact of concussions on neuromuscular control and anterior cruciate ligament injury risk in female soccer players: Mechanisms and prevention—A narrative review. J. Clin. Med..

[16] Parr J.V., Uiga L., Marshall B., Wood G. (2023). Soccer heading immediately alters brain function and brain-muscle communication. Front. Hum. Neurosci..

[17] Shamloo F., Kon M., Ritter E., Sereno A.B. (2023). Quantifying the magnitude and longevity of the effect of repetitive head impacts in adolescent soccer players: Deleterious effect of long headers extend beyond a month. Neurotrauma Rep..

[18] Chmielewski T.L., Tatman J., Suzuki S., Horodyski M., Reisman D.S., Bauer R.M., Clugston J.R., Herman D.C. (2021). Impaired motor control after sport-related concussion could increase risk for musculoskeletal injury: Implications for clinical management and rehabilitation. J. Sport Health Sci..

[19] Dolman K.E., Staines R.S., Mughal S., Brown K.E., Meehan S.K., Staines W.R. (2025). Long-term effects of concussion on attention, sensory gating and motor learning. Exp. Brain Res..

[20] Eagle S.R., Kontos A.P., Pepping G.-J., Johnson C.D., Sinnott A., LaGoy A., Connaboy C. (2020). Increased risk of musculoskeletal injury following sport-related concussion: A perception–action coupling approach. Sports Med..

[21] Hayes K.D., Khan M.E., Graham K.R., Staines W.R., Meehan S.K. (2025). Persistent adaptations in sensorimotor interneuron circuits in the motor cortex with a history of sport-related concussion. Exp. Brain Res..

[22] Howell D.R., Lynall R.C., Buckley T.A., Herman D.C. (2018). Neuromuscular control deficits and the risk of subsequent injury after a concussion: A scoping review. Sports Med..

[23] Lempke L.B., Lynall R.C. (2025). The state of the science for potential contributors to musculoskeletal Injury following concussion: Mechanisms, gaps, and clinical considerations. Musculoskelet. Sci. Pract..

[24] Wilke J., Groneberg D.A. (2022). Neurocognitive function and musculoskeletal injury risk in sports: A systematic review. J. Sci. Med. Sport.

[25] Burcal C.J., Needle A.R., Custer L., Rosen A.B. (2019). The effects of cognitive loading on motor behavior in injured individuals: A systematic review. Sports Med..

[26] Hutchison M., Comper P., Mainwaring L., Richards D. (2011). The influence of musculoskeletal injury on cognition: Implications for concussion research. Am. J. Sports Med..

[27] Kiefer A.W., DiCesare C., Nalepka P., Foss K.B., Thomas S., Myer G.D. (2018). Less efficient oculomotor performance is associated with increased incidence of head impacts in high school ice hockey. J. Sci. Med. Sport.

[28] Kung S.M., Suksreephaisan T.K., Perry B.G., Palmer B.R., Page R.A. (2020). The effects of anticipation and visual and sensory performance on concussion risk in sport: A review. Sports Med. Open.

[29] Wilkerson G.B., Colston M.A., Acocello S.N., Hogg J.A., Carlson L.M., Lunt E.N., Palk A.R., Smith H.N. (2025). Introspective ratings of biopsychosocial status are associated with sport-related injuries. J. Athl. Train..

[30] Cardoso F.d.S.L., Neves J.A., Roca A., Teoldo I. (2021). The association between perceptual-cognitive processes and response time in decision making in young soccer players. J. Sports Sci..

[31] Gallivan J.P., Chapman C.S., Wolpert D.M., Flanagan J.R. (2018). Decision-making in sensorimotor control. Nat. Rev. Neurosci..

[32] Gokeler A., Tosarelli F., Buckthorpe M., Della Villa F. (2024). Neurocognitive errors and noncontact anterior cruciate ligament injuries in professional male soccer players. J. Athl. Train..

[33] Li J., Cao Y., Ou S., Jiang T., Wang L., Ma N. (2024). The effect of total sleep deprivation on working memory: Evidence from diffusion model. Sleep.

[34] Luo J., Hao C., Ma N., Wang L. (2024). Sleep deprivation affects interference control: A diffusion model analysis. J. Exp. Psychol. Hum. Percept. Perform..

[35] Haskell B., Eiler A., Essien H. (2025). Sleep quality and cognitive skills impact neurocognitive function and reduce sports-related injury risk. Arthrosc. Sports Med. Rehabil..

[36] Ratcliff R., Van Dongen H.P. (2011). Diffusion model for one-choice reaction-time tasks and the cognitive effects of sleep deprivation. Proc. Natl. Acad. Sci. USA.

[37] Viegas F., Ocarino J.M., de Sousa Freitas L., Pinto M.C., Facundo L.A., Amaral A.S., Silva S., de Mello M.T., Silva A. (2022). The sleep as a predictor of musculoskeletal injuries in adolescent athletes. Sleep Sci..

[38] von Rosen P., Frohm A., Kottorp A., Fridén C., Heijne A. (2017). Multiple factors explain injury risk in adolescent elite athletes: Applying a biopsychosocial perspective. Scand. J. Med. Sci. Sports.

[39] Raikes A.C., Athey A., Alfonso-Miller P., Killgore W.D., Grandner M.A. (2019). Insomnia and daytime sleepiness: Risk factors for sports-related concussion. Sleep Med..

[40] Riegler K.E., Guty E.T., Thomas G.A., Bradson M.L., Arnett P.A. (2023). Prospective implications of insufficient sleep for athletes. J. Athl. Train..

[41] Palmer C.A., Alfano C.A. (2017). Sleep and emotion regulation: An organizing, integrative review. Sleep Med. Rev..

[42] Brett B.L., Meier T.B., Savitz J., Guskiewicz K.M., McCrea M.A. (2021). Research letter: Sleep mediates the association between prior concussion and depressive symptoms. J. Head. Trauma Rehabil..

[43] Van Bortel K.M., Hamill K.E., Goeckner B.D., Mayer A.R., Brett B.L., Meier T.B. (2024). The relationship between multiple concussions and multidimensional sleep quality in collegiate-aged, active athletes. Sleep Health.

[44] Prather A.A., Grandner M.A. (2019). Sleep, stress, and immunity. Sleep and Health.

[45] Ao Y., Klar P., Catal Y., Wang Y., Northoff G. (2025). Infra-slow scale-free dynamics modulate the connection of neural and behavioral variability during attention. Commun. Biol..

[46] Yamashita A., Rothlein D., Kucyi A., Valera E.M., Germine L., Wilmer J., DeGutis J., Esterman M. (2021). Variable rather than extreme slow reaction times distinguish brain states during sustained attention. Sci. Rep..

[47] Paludo A., Lipčák A., Parpa K., Kyprianidou E., Avraamides M. (2026). The effect of virtual and augmented reality training on soccer players: A systematic review of cognitive-motor performance. Biol. Sport.

[48] Tugwell P., Knottnerus J.A. (2015). Is the ‘Evidence-Pyramid’now dead?. J. Clin. Epidemiol..

[49] Rodu J., DeJong Lempke A.F., Kupperman N., Hertel J. (2024). On leveraging machine learning in sport science in the hypothetico-deductive framework. Sports Med. Open.

[50] Carpi M. (2025). The Pittsburgh sleep quality index: A brief review. Occup. Med..

[51] Dietch J.R., Taylor D.J., Sethi K., Kelly K., Bramoweth A.D., Roane B.M. (2016). Psychometric evaluation of the PSQI in US college students. J. Clin. Sleep Med..

[52] Patridge E.F., Bardyn T.P. (2018). Research electronic data capture (REDCap). J. Med. Libr Assoc..

[53] Wilkerson G.B., Gullion A.J., McMahan K.L., Brooks L.T., Colston M.A., Carlson L.M., Hogg J.A., Acocello S.N. (2025). Perceptual decision efficiency Is modifiable and associated with decreased musculoskeletal injury risk among female college soccer players. Brain Sci..

[54] Wilkerson G.B., Mether K.S., Perrin Z.A., Emberton S.L., Carlson L.M., Hogg J.A., Acocello S.N. (2024). Perceptual response training for reduction of injury risk among high school girls’ soccer players. Brain Sci..

[55] Wilkerson G.B., Fleming L.R., Adams V.P., Petty R.J., Carlson L.M., Hogg J.A., Acocello S.N. (2024). Assessment and training of perceptual-motor function: Performance of college wrestlers associated with history of concussion. Brain Sci..

[56] Lakens D. (2013). Calculating and reporting effect sizes to facilitate cumulative science: A practical primer for *t*-tests and ANOVAs. Front Psychol..

[57] Fiel Peres F. (2026). Effect sizes for nonparametric tests. Biochem Med..

[58] Alba A.C., Agoritsas T., Walsh M., Hanna S., Iorio A., Devereaux P., McGinn T., Guyatt G. (2017). Discrimination and calibration of clinical prediction models: Users’ guides to the medical literature. JAMA.

[59] Olivier J., May W.L., Bell M.L. (2017). Relative effect sizes for measures of risk. Commun. Stat. Theory Methods.

[60] Lee L.Y., Ziminski J.J., Frangou P., Karlaftis V.M., Wang Y., Bernhardt B., Warrier V., Bethlehem R.A.I., Kourtzi Z. (2025). Neurogenetic phenotypes of learning-dependent plasticity for improved perceptual decisions. Commun. Biol..

[61] Ting C.-C., Gluth S. (2024). Unraveling information processes of decision-making with eye-tracking data. Front Behav. Econ..

[62] Ivanov V., Manenti G.L., Plewe S.S., Kagan I., Schwiedrzik C.M. (2024). Decision-making processes in perceptual learning depend on effectors. Sci. Rep..

[63] Sorrentino G., Edwards M.G., Baldini N., Mustile M., Lejeune T., Everard G. (2025). REAsmash-ET: A methodological framework for combined cognitive and motor assessment through eye-tracking and kinematic metrics in immersive VR search-and-reach task. J. Neuroeng. Rehabil..

[64] Chen J., Oddson B., Skrinar H. (2022). Incremental effects of subsequent concussions on cognitive symptoms in the sport concussion assessment tool. Clin. J. Sport Med..

[65] Ledreux A., Pryhoda M.K., Gorgens K., Shelburne K., Gilmore A., Linseman D.A., Fleming H., Koza L.A., Campbell J., Wolff A. (2020). Assessment of long-term effects of sports-related concussions: Biological mechanisms and exosomal biomarkers. Front Neurosci..

[66] van Vugt M.K., Simen P., Nystrom L., Holmes P., Cohen J.D. (2014). Lateralized readiness potentials reveal properties of a neural mechanism for implementing a decision threshold. PLoS ONE.

[67] Hammer J., Kajsova M., Kalina A., Krysl D., Fabera P., Kudr M., Jezdik P., Janca R., Krsek P., Marusic P. (2024). Antagonistic behavior of brain networks mediated by low-frequency oscillations: Electrophysiological dynamics during internal–external attention switching. Commun. Biol..

[68] Zhang H., Yang S.Y., Qiao Y., Ge Q., Tang Y.Y., Northoff G., Zang Y. (2022). Default mode network mediates low-frequency fluctuations in brain activity and behavior during sustained attention. Hum. Brain Mapp..

[69] Duffy J.S., Bellgrove M.A., Murphy P.R., O’Connell R.G. (2025). Disentangling sources of variability in decision-making. Nat. Rev. Neurosci..

[70] Garrett D.D., Samanez-Larkin G.R., MacDonald S.W., Lindenberger U., McIntosh A.R., Grady C.L. (2013). Moment-to-moment brain signal variability: A next frontier in human brain mapping?. Neurosci. Biobehav. Rev..

[71] Perri R.L., Di Russo F. (2017). Executive functions and performance variability measured by event-related potentials to understand the neural bases of perceptual decision-making. Front Hum. Neurosci..

[72] Waschke L., Kloosterman N.A., Obleser J., Garrett D.D. (2021). Behavior needs neural variability. Neuron.

[73] Seideman J.A., Stanford T.R., Salinas E. (2018). Saccade metrics reflect decision-making dynamics during urgent choices. Nat. Commun..

[74] Imaoka Y., Flury A., De Bruin E.D. (2020). Assessing saccadic eye movements with head-mounted display virtual reality technology. Front Psychiatr..

[75] Pierce J.E., Clementz B.A., McDowell J.E., Klein C., Ettinger U. (2019). Saccades: Fundamentals and neural mechanisms. Eye Movement Research: An Introduction to Its Scientific Foundations and Applications.

[76] McDowell J.E., Dyckman K.A., Austin B.P., Clementz B.A. (2008). Neurophysiology and neuroanatomy of reflexive and volitional saccades: Evidence from studies of humans. Brain Cogn..

[77] Gibaldi A., Sabatini S.P. (2021). The saccade main sequence revised: A fast and repeatable tool for oculomotor analysis. Behav. Res. Methods.

[78] Tan C.H., Moss H.E., England J.D. (2025). Saccades. Encyclopedia of the Neurological Sciences.

[79] Aksum K.M., Brotangen L., Bjørndal C.T., Magnaguagno L., Jordet G. (2021). Scanning activity of elite football players in 11 vs. 11 match play: An eye-tracking analysis on the duration and visual information of scanning. PLoS ONE.

[80] Zhang Q., Huang Z., Li L., Li S. (2022). Visual search training benefits from the integrative effect of enhanced covert attention and optimized overt eye movements. J. Vis..

[81] Appelbaum L.G., Lochhead L., Feng J., Erickson G., Liu S., Laby D.M. (2025). Limited evidence is NOT no evidence: A rebuttal to Fransen, 2024. Sports Med..

[82] Fransen J. (2024). There is no supporting evidence for a far transfer of general perceptual or cognitive training to sports performance. Sports Med..

[83] Irwin M.R., Opp M.R. (2017). Sleep health: Reciprocal regulation of sleep and innate immunity. Neuropsychopharmacology.

[84] Lezama E., Treviño M. (2025). Visuomotor processing in the superior colliculus. Acad. Neurosci. Brain Res..

[85] He B.J., Zempel J.M. (2013). Average is optimal: An inverted-U relationship between trial-to-trial brain activity and behavioral performance. PLoS Comput Biol..

[86] Hirshberg M.J., Dahl C.J., Bolt D.M., Davidson R.J., Goldberg S.B. (2025). Psychological mediators of reduced distress: Preregistered analyses from a randomized controlled trial of a smartphone-based well-being training. Clin. Psychol. Sci..

[87] Kral T.R., Imhoff-Smith T., Dean D.C., Grupe D., Adluru N., Patsenko E., Mumford J.A., Goldman R., Rosenkranz M.A., Davidson R.J. (2019). Mindfulness-based stress reduction-related changes in posterior cingulate resting brain connectivity. Soc. Cogn. Affect Neurosci..

[88] Cao J., Zhang S. (2014). Multiple comparison procedures. JAMA.

[89] Rothman K.J. (1990). No adjustments are needed for multiple comparisons. Epidemiology.

[90] Bland J.M., Altman D.C. (2000). The odds ratio. BMJ.

[91] Wilkerson G.B., Lansey J.C., Noblett C.N., Sarris C.E. (2023). Test-retest reliability of immersive virtual reality measures of perceptual-motor performance. Percept. Mot. Ski..

[92] Mah C.D., Kezirian E.J., Marcello B.M., Dement W.C. (2018). Poor sleep quality and insufficient sleep of a collegiate student-athlete population. Sleep Health.

